# Plant Extracts Mediated Metal-Based Nanoparticles: Synthesis and Biological Applications

**DOI:** 10.3390/biom12050627

**Published:** 2022-04-24

**Authors:** Jerry O. Adeyemi, Ayodeji O. Oriola, Damian C. Onwudiwe, Adebola O. Oyedeji

**Affiliations:** 1 Department of Chemical and Physical Sciences, Faculty of Natural Sciences, Walter Sisulu University, Mthatha 5099, South Africa; aooriola@gmail.com; 2 Department of Chemistry, Faculty of Natural and Agricultural Science, North-West University, Private Bag X2046, Mmabatho 2735, South Africa; damian.onwudiwe@nwu.ac.za; 3 Material Science Innovation and Modelling (MaSIM) Research Focus Area, Faculty of Natural and Agricultural Sciences, Mafikeng Campus, North-West University, Private Bag X2046, Mmabatho 2735, South Africa

**Keywords:** metal-based nanoparticles, plant extracts, phytochemicals, metabolite, biological properties

## Abstract

The vastness of metal-based nanoparticles has continued to arouse much research interest, which has led to the extensive search and discovery of new materials with varying compositions, synthetic methods, and applications. Depending on applications, many synthetic methods have been used to prepare these materials, which have found applications in different areas, including biology. However, the prominent nature of the associated toxicity and environmental concerns involved in most of these conventional methods have limited their continuous usage due to the desire for more clean, reliable, eco-friendly, and biologically appropriate approaches. Plant-mediated synthetic approaches for metal nanoparticles have emerged to circumvent the often-associated disadvantages with the conventional synthetic routes, using bioresources that act as a scaffold by effectively reducing and stabilizing these materials, whilst making them biocompatible for biological cells. This capacity by plants to intrinsically utilize their organic processes to reorganize inorganic metal ions into nanoparticles has thus led to extensive studies into this area of biochemical synthesis and analysis. In this review, we examined the use of several plant extracts as a mediating agent for the synthesis of different metal-based nanoparticles (MNPs). Furthermore, the associated biological properties, which have been suggested to emanate from the influence of the diverse metabolites found in these plants, were also reviewed.

## 1. Introduction

Nanotechnology has continued to garner extensive attention over the past few decades due to its wide and useful applications in biology [1], biotechnology [2], energy [3], information technology [4], environmental remediations [5,6], and medical technology [7,8]. This technology provides the platform to work at the atomic, molecular, and supramolecular level of material, within the range of 1–100 nm, to create, understand and apply the materials’ structures, with fundamentally new properties, due to the newly formed structures [9]. Generally, all-natural and man-made systems are known to possess a first-level organization at a nanoscale level with a well-defined fundamental property, such as in nanocrystals, nanotubes, and nanomotors [9]. Nanotechnology describes the pattern in which atoms hierarchically assemble and disassemble into objects, along various scales of length [9]. Consequently, several biobased nanomaterials and devices have been fabricated using the platform of nanotechnology due to the ability to fine-tune several intrinsic properties, responses, and functions to achieve a more desirable outcome than their bulk counterparts [8,9]. This possibility to make new advanced products from already established ones, with better functions and characteristics, have created the impetus for the continuous interest in this area of research, especially in the field of nanomedicine. 

The term “nanomedicine” has been used to generally describe the science and technology of preventing, diagnosing, and treatment of different forms of diseases using nanomaterials that have been carefully engineered to perform these functions [10]. Great expectations have been accorded to the use of nanotechnology due to the abundant opportunities to impact human lives positively. These opportunities, especially in many biological processes, have emerged because most of their processes proceed at a nanometre scale within smaller molecular units, such as amino acids, DNA, proteins, and cellular membranes [8]. Therefore, the miniaturization of biomedical products seems to be the future of biomedicine. As such, one notable example of nanomaterials that has received considerable attention in recent years and has been widely used is metal-based nanomaterials. 

The formation of metal-based nanoparticles is not a recent technology because many organisms synthesize them during heavy metal detoxification and their vastness in many technologies has been widely applied in recent decades [11]. Metal-based nanomaterials are the most synthesized and the most useful of the inorganic nanoparticles (NPs) which represent a promising solution in the field of biology and medicine [12,13,14,15,16]. Their recent surge in literature reports on the numerous synthetic documentations of these types of nanoparticles stems from their increasing application, which takes advantage of their enhanced physical, optical, biological, and magnetic properties [17]. The most synthesized and used in this class are those of transition metal origins, such as gold, silver, zinc, iron, and copper [18,19,20]. Generally, transition metal-based nanoparticles are regarded as the best candidate for metal-based nanomaterials due to the presence of partially filled d-orbitals which make them more redox-active (easier to reduce to zero-valent atoms), a feature that facilitates their nanoparticle aggregation [18]. Their vastness has consequently aroused much research interest, which has led to the continuous search and discovery of new materials of varying compositions, eco-friendly methods, and applications [12]. Specifically, those prepared using phytochemicals from plant materials have been of significant interest due to their usage for invasive applications in medicine [21,22]. Thus, different synthetic routes have been designed for the eco-friendly preparations of these materials from their corresponding metal salts [21]. In this review, the importance of the different plant metabolites (phytochemicals) in the synthesis of metal-based nanoparticles using different plant materials and their respective applications as biological agents, have been briefly discussed. 

## 2. Phytochemical-Induced Synthesis of Metal-Based Nanoparticles 

**Figure 1:**
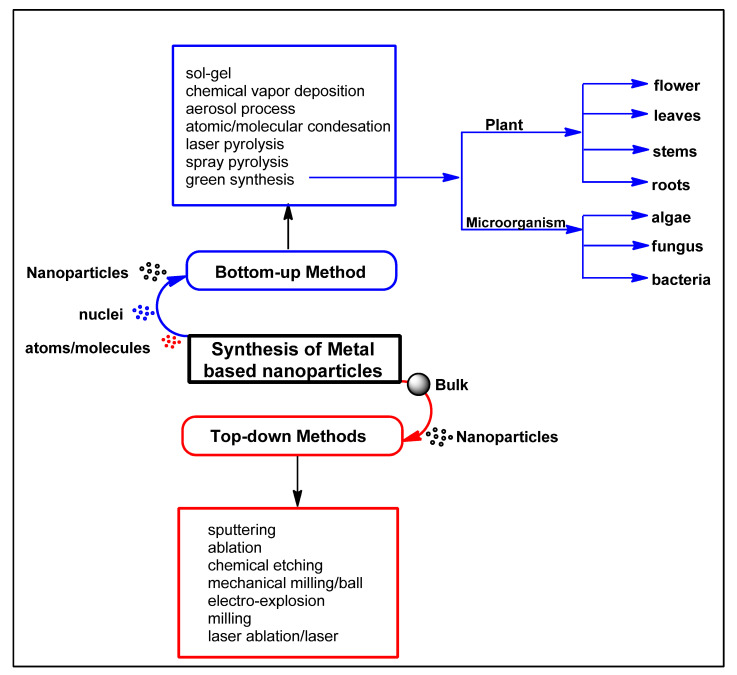
Different reported synthetic approaches for metal-based nanoparticles. Redrawn from [21], with permission from Elsevier (Copyright 2022).

Depending on the proposed applications, different synthetic methods have been used to prepare these nanomaterials (with unique and interesting properties) for applications in sensing, catalysis, electronics, photonics, biomedicine, and many, etc. [18]. In general, two synthesis routes: top-down and bottom-up are used in materials synthesis as shown in Figure 1 [23,24]. However, the associated toxicity and environmental concerns involved in some of these methods due to the use of toxic reducing substances, organic solvents, and stabilizers (for a reduction in metals and prevention of agglomeration of the colloids) have led to the desire for a clean, reliable, environmentally friendly, and biologically appropriate approaches. Thus, the emergence of green synthetic approaches that use plant extract, microorganisms, and some marine algae [21] (Figure 1) as eco-friendly approaches, is being used in the preparations of nanoparticles (especially for those materials used for invasive applications in medicine) [21]. Many eco-friendly methods, which involve the use of different plant materials (containing various arrays of phytochemicals), microorganisms, and some marine algae, as seen in Figure 1, have been well received in recent years [21].

The advantages of these biogenic methods of preparation are not only limited to their eco-friendliness when compared to the other methods, these methods are also cheap and useful in the preparation of many nanoparticles, which are usually free from contaminants and possess a well-defined morphology and size [21].

The use of plant materials as a reducing agent for different metal ions dates to the early 1900s, even though the mechanism of action and what was responsible for this reduction was not clear at the time [21]. The ease of preparation and ability to act as a reducing agent made their usage attractive, which in turn gave rise to increased attention within the last few decades [21,25,26]. Plant-mediated methods have proven to proceed via the rapid intra or extracellular processes [25]. Most phytochemicals, such as terpenes, alkaloids, saponins, phenols, and alcohol present in plant materials, carry out the reduction processes of these metal salts [27]. Most of these phytochemicals are found in different parts of plants, such as flowers, fruits, stems, leaves, and roots, resulting in several reports on the synthesis of plant-mediated metal-based nanoparticles [25,27]. Some factors are known to affect the preparation and properties of the prepared nanoparticles, which include the type of plant extract used, its concentration, the pH of the medium, the concentration of the metal salt, contact time, and temperature. All these factors have been reported to affect the rate, properties, and quantity of the prepared nanoparticles [28]. Generally, the method involves the mixing of the extract of interest with an aqueous solution of the metal salt at room temperature. Although different temperatures have been reported, many silver and gold nanoparticles have been prepared at room temperature [28]. 

**Figure 2:**
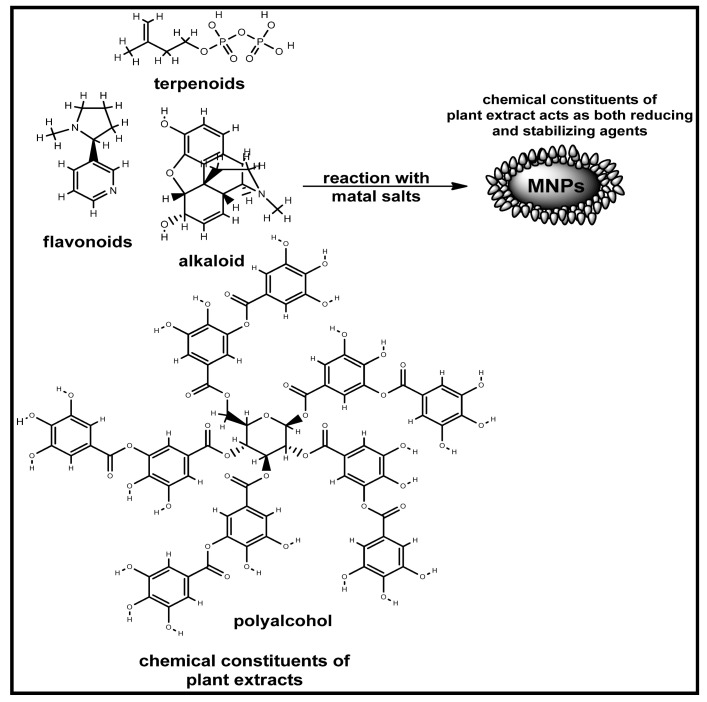
Selected possible constituents of plant extract responsible for the bio-reduction of the metal salts during the synthetic preparation. Redrawn from [34,35], with permission from Elsevier (Copyright 2022).

Silver nanoparticles (AgNPs) are one of the notable metal nanoparticles that have been extensively studied over the years. This is due to its fascinating, unique physical and chemical properties, which have led to their diverse application in many fields [29]. Although several other noble metals have also been widely used for several purposes, AgNPs have received a lot of attention in the diagnosis and treatment of several diseases, including cancer [29]. Hence, the interest in its various synthetic routes cannot be overemphasized. Narayanan and Sakthivel have reported the synthesis of AgNPs using the leaf extract of *Coleus amboinicu*, and the obtained morphology, such as shape and size, were influenced by the concentration of the extract mixed with the silver salt [30]. The rhizome extract of *Discorea batatas* has also been used to prepare AgNPs with good antimicrobial properties against the yeasts *Saccharomyces cerevisiae* and *C. Albicans*, according to Nagajyothi and Lee [31]. Ahmad et al. [32] reported the synthesis of AgNPs using the plant extracts of *D. Trifolium.* The reduction of silver ions in AgNPs was reported to be brought about by the presence of ascorbic acid found in the extract, as well as the presence of NAD+ and H^+^ ions [32]. In a similar vein, the leaf extract of *Datura Metel* has been used to produce very stable AgNPs of 16–40 nm [33]. The reduction was attributed to the presence of some phytochemicals, such as amino acids, enzymes, alcoholic compounds, alkaloids, and polysaccharides [33]. Figure 2 shows the probable constituents of some plant extracts responsible for the reduction and the likely stabilization of the AgNPs [21,34,35].

Another notable noble metal nanoparticle that has remained of interest in recent years, despite their long history, is gold nanoparticles. The origin of gold nanoparticles (AuNPs) dates to the ancient time when they solely served aesthetics and herbal purposes for the decoration of vessels and for curing diverse diseases [28]. Its modern usage, however, started some few centuries back when Michael Faraday found that the nanoform of gold, in its colloidal state, possessed some properties different from its bulk state [36]. These unique properties, in the nanoform, have led to its diverse applications, especially in biology, which has led to the continuous desire to prepare it, albeit in an eco-friendly process. For instance, Daisy and Saipriya (2012) utilized *Cassia fistula* bark to prepare AuNPs of a particle size between 55 and 98 nm. The AuNPs were found to show a better hypoglycaemic property, used in the treatment of diabetes, in experimental rats than the pure plant extract [37,38]. Using chrysanthemum and tea beverages, Liu et al. [34] similarly synthesized and examined the antioxidant properties of AuNPs. Pulp of sugar has also been used in making Au nano-rods and wires at room temperatures and varying pH [39]. 

Although many procedures and routes for making MNPs with useful properties, such as shape and size have now been well established in the literature, nevertheless, the limited properties of a single-metal nanoparticle have restricted their application to several other fields. One of such ways in which properties of this material can be fine-tuned involves compositing with other materials that bear a different functional property to parent materials. Consequently, the design of metal nanoparticles with two or more distinct metals is now being reported extensively, as they possess fascinating properties which are sometimes extensively different from the corresponding single-metal nanoparticles [24,40,41]. An example of a property that has been studied, due to its ability to fine-tune the optical properties of a material with greater versatility, is the shape of the material [40]. Thus, controlling properties, such as size, shape, and composition are very important in tailoring the functions and application of nanomaterials [40,42,43]. In multifunctional nanoparticles, using Au-alloy particles as a case study, properties, such as plasmonic properties, optical properties, and magnetic properties have been enhanced, according to several reports [44,45,46]. These enhanced properties emerge from the new structural and electronic effects brought about by the combination of these metals [44,45,46]. 

**Figure 3:**
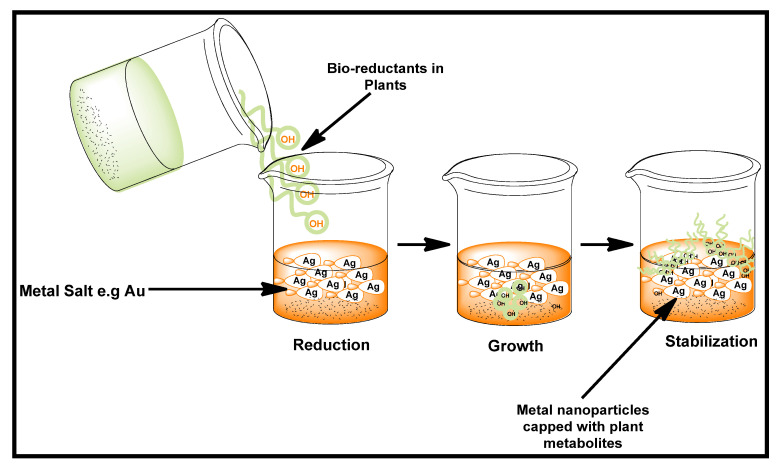
Green synthesis scheme for Au, Ag, and Au–Ag nanoparticles. Redrawn from [50], with permission from Elsevier (Copyright 2022).

The application of bimetallic nanoparticles (BMNPs) has been widely reported for enhanced biological potential [40,41,47]. An example that has been widely studied in recent times is gold–silver nanoparticles. Shankar et al., using the leaf extracts of *P. graveolens* and *Azadirachta indica*, reported the formation of a bimetallic Ag–Au core–shell [48,49]. The reduction and stabilization of the BMNPs were attributed to the presence of reducing sugars. The formation of Ag–Au BMNPs using different concentrations of Kei apple (*Dovyalis caffra*) fruit extract has been reported by our group. The proposed scheme of preparation is shown in Figure 3. The as-prepared BMNPs were found to show a more promising cytotoxic activity than their respective single-metal NPs. The BMNPs synthesized using a higher concentration of the fruit extract demonstrated the best cytotoxicity against the breast cancer MCF7 cell line [50]. 

In the last few decades, metal oxide nanoparticles (MONPs) have been widely reported using the green synthesis approach [51]. Notable examples include zinc oxide (ZnO), copper oxide (CuO), nickel oxide (NiO), magnesium oxide (MgO), iron oxide (Fe_3_O_2_) titanium dioxide (TiO_2_), and cerium oxide (CeO_2_) nanoparticles. These nanomaterials have been widely applied in many areas of science, including biology [2,28,52]. Their ease of preparation stems from the fact that most thermal elements possess the capacity to exist in a wide range of oxides while adopting a vast array of structural geometries with a structure that can exhibit electrical characteristics [51]. Metal oxide nanoparticles possess a distinct opto-electrical property due to their localized surface plasmon resonance features [53]. This feature has made them highly attractive in the field of medicine, most especially in biomedical therapeutics, bio-imaging, and bio-sensing agents [53]. They have garnered much attention in the past few years due to their application as implant agents, neurochemical monitoring agents, and in the diagnosis and treatment of cancer [53]. Titanium dioxide (TiO_2_), for instance, has become a choice material for several medical implants due to its excellent biocompatible surface, which fosters cell attachment and proliferation. Likewise, cerium oxide (CeO_2_) nanoparticles have shown useful antioxidant, redox, and auto-catalytic properties [53]. Most of these materials, such as those reported in our group, using a South African indigenous plant called Kei apple [27], have been extensively prepared using different plant extracts due to the numerous applications. Different metals and bimetallic nanoparticles, as well as biologically relevant metal oxide nanoparticles derived from using extracts of several plant materials, have been summarized in Table 1.

### 2.1. Possible Mechanism for Synthesis of Nanoparticles Using Plant Extract

**Figure 4:**
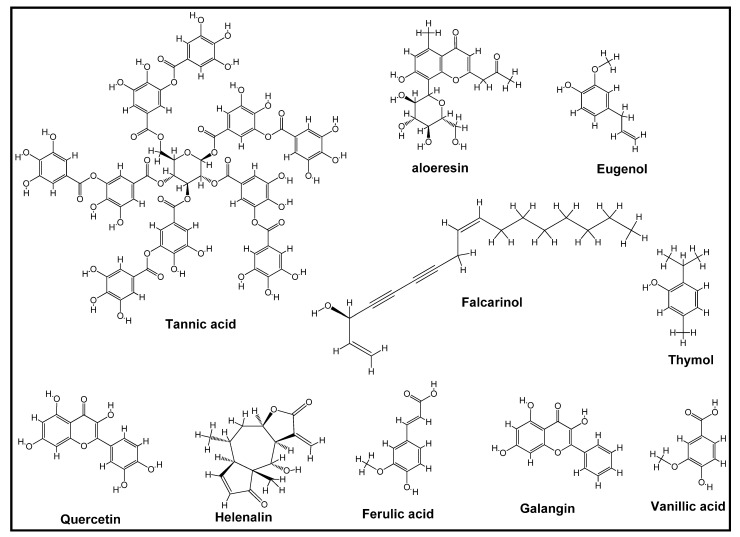
Structures of some examples of secondary metabolites in plant.

Despite several reported studies on the plant-mediated synthesis of nanoparticles, only very few pieces of literature are available on the mechanism of their synthesis [73,74]. The understanding of the mechanism involved in the synthesis of nanoparticles using plant extract is important due to the diverse emerging applications of this class of materials in various fields of life, such as medicine. Hence, it is important to explore different synthesis approaches so that properties, such as crystallinity, shape, size, and disparity, can be easily controlled. Although there are a few proposed mechanisms for the synthesis of metal-based nanoparticles since the 1990s, a newly proposed mechanism has now emerged for the biosynthesis of nanomaterials, especially those from plant extracts [74,75]. Secondary metabolites found in most plants, such as sugar, terpenoids, polyols, alkaloids phenolic acids, and proteins, play important roles in the synthesis of metal-based nanoparticles [76]. Some representative examples of these metabolites have been presented in Figure 4. 

**Figure 5:**
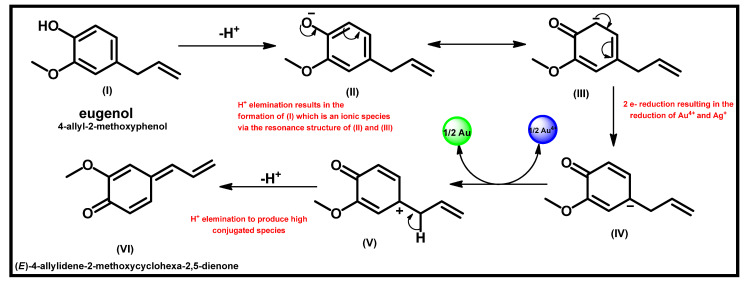
A schematic representation of the mechanism of reduction in Au^4+^ and Ag^+^ using plant extract containing a phytochemical like eugenol. Redrawn from [77], with permission from Springer Nature (Copyright 2022).

Singh et al. [77] have highlighted the mechanism of the formation using the FTIR spectroscopic study of extracts of *C. zeylanicum*. In this study, the Fourier transform infrared (FTIR) spectroscopy result suggested that the reduction process was achieved by the presence of terpenoids, (a class of different organic polymers that possess a five-carbon isoprene chain) due to their strong affinity for metal ions. In this study, eugenol, a type of terpenoid, was suggested to be responsible for the reduction of the silver and gold salts into their respective silver and gold nanoparticles [77]. The study suggested (using only FTIR analysis) that the resonance structure that precedes the formation of the metal-based nanoparticles emerges from proton abstraction from eugenol in the presence of an –OH group [77] (see Figure 5 for details).

Similarly, flavonoids, a secondary metabolite in plants, have also been reported to play an active part in the reduction and chelation of metal ions. This was attributed to the release of hydrogen ions during the tautomeric transformation of flavonoids (from enol form to keto form) [78,79]. Flavonoids belong to a class of polyphenolic compounds that comprise different classes, such as flavones, flavanones, flavonoids, isoflavonoids, chalcones, and anthocyanins. Flavonoids were thought to be involved in the *Ocimum basilicum* and *Mangifera indica* leaf mediated synthesis of silver nanoparticles (AgNPs) [78,79]. They were thought to be the key players in the reduction process of Ag ions to Ag nanoparticles [78]. The ketone and carboxylic acid groups present in the flavonoids have been identified as the major players. In another example, quercetin, an example of flavonoids, was identified as a strong chelating agent due to the carbonyl and hydroxyl groups present in its C3 and C5 positions. This class of flavonoid is responsible for the chelation of some metal ions, including Al^3+^, Co^2+^, Pb^2+^, Fe^2+^, Fe^3+^, and Cu^2+^ [73,80]. Furthermore, the number of polyphenols present in the extract has been reported to play a major role in the observed size and distribution of the prepared metal nanoparticles [79]. Other reports involving the bimetallic synthesis of metal nanoparticles have identified terpenoids and flavonoids as the major players responsible for the reduction and stabilization [81]. The possible synthetic mechanism and stabilization for most noble metal nanoparticles have been reported by Song et al. [82] using *Magnolia Kobus* leaf extracts. It was reported that the principal functional group responsible for the reduction and stabilization of the prepared gold nanoparticles are ketones, amines, aldehydes, alcohols, and carboxylic acid. Their bimetallic silver and gold nanoparticles, using the same leaf extract, were prepared by Begum et al. [83], and it was concluded using cyclic voltammetry and FTIR spectroscopy that the flavonoids or polyphenols were the key players in this case [83].

**Figure 6:**
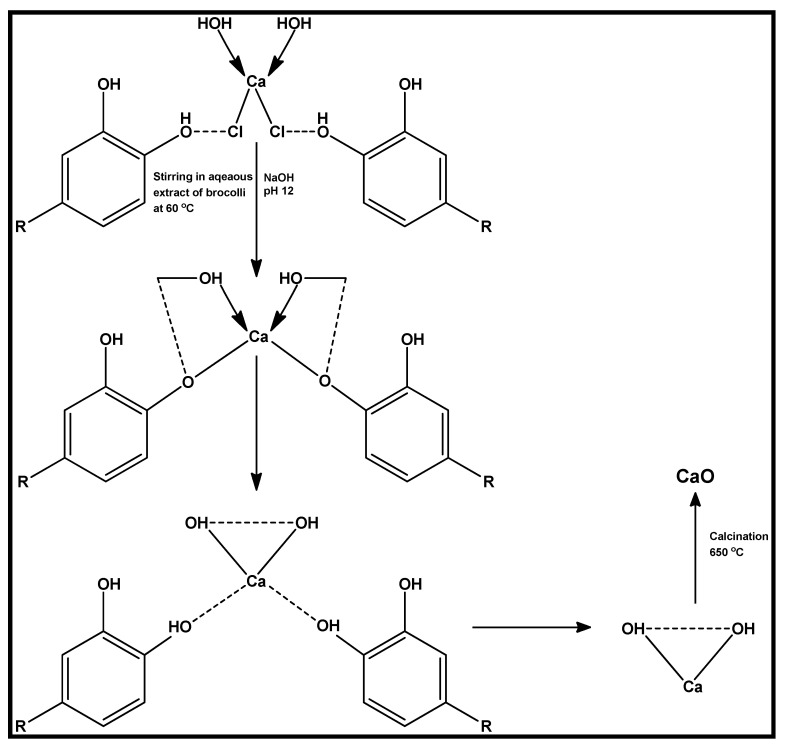
Suggested mechanism of the synthesis of CaO nanoparticles mediated using aqueous broccoli extract. Redrawn from [84], with permission from John Wiley and Sons (Copyright 2022).

Other mechanisms involving the synthesis of metal-based nanoparticles, such as metal oxide nanoparticles, have also been proposed in the literature. Osuntokun et al. [75], using broccoli extracts, suggested polyphenols, and flavonoids as the main reducing secondary metabolite in the reported CaO nanoparticles. A reaction mechanism was proposed (see Figure 6), using quercetin, a flavonoid, as a representative of the active phytochemicals in broccoli. From the proposed scheme, it was suggested that the flavonoid binds to the metal salts, thereby reducing it to a metal ion, which then reacts with the OH^-^ within the quercetin. This consequently leads to the formation of Ca(OH)_2_ which upon drying and calcination produces CaO nanoparticles [84]. The phenolic metabolites were confirmed with FTIR and were suggested to act as both reducing and stabilizing agents [84]. This was also similar to our earlier report on the synthesis of ZnO nanoparticles using the aqueous extract of Kei Apple fruits. However, in this case, the phytochemicals, such as salicylic acid, m-hydroxybenzoic acid, vanillic acid, gallic, and catechins, were the principal reducing agents [27]. 

Generally, there are three major requirements for the synthesis of this class of materials via the plant extract route: the reducing agent, stabilizing agent, and solvent medium needed for stabilizing the desired nanoparticle [85]. The use of biological material for the synthesis of nanomaterials is generally regarded as a green process because they possess the potential to reduce and stabilize the desired nanoparticles. Furthermore, most of these plant-based synthetic processes can proceed in an aqueous medium instead of conventional organic solvents. Three reaction regimes have been proposed to occur during this biosynthetic process, including a short incubation period, a growth phase, and a termination period [86]. The reduction and the nucleation phases, which usually bring about the large yield of the small size particles, are faster than the growth phase of the particles. Furthermore, reports that metal ions have the potential to also act as biomass through the formation of an ionic bond with the bio-organic reducing agents, such as flavonoids and terpenoids, in the absence of other strong ligands, have been made [86]. Moreover, the absorption of reducing agents on these nanoparticles’ surfaces has been attributed to the presence of π-electrons and the carbonyl groups present within their molecular structures [73].

### 2.2. Determination of Physicochemical Properties of Metal-Based Nanomaterials

Generally, most materials synthesized in the nanometre regime are intermediates between the bulk and the isolated small molecules [87]. Their unique physicochemical properties in comparison to their bulk counterparts, such as shape, size, composition, surface properties, solubility, stability, molecular weight, and purity, are very important in their physiological interactions and may provide some useful benefits in their application as a therapeutic agent [87,88]. The impact of these properties on their physiological behaviours plays a major role in influencing their diagnostic efficacy or therapeutic potential in nanomedicines. It is therefore pertinent to understand how the different physicochemical characteristics affect their biodistribution and behaviour, which in this report, are mostly at cellular levels [87,89,90].

There are different techniques used for the characterization of nanoparticles, and more techniques continue to emerge for the purpose of understanding the properties of nanomaterials. The most used techniques in characterizing, as well as ascertaining the common properties of the prepared nanoparticles include but are not limited to the following: powder X-ray diffraction (XRD), Fourier transform infrared spectroscopy (FTIR), UV–visible spectrophotometry, transmission electron microscopy (TEM), scanning electron microscopy (SEM), energy dispersive spectroscopy (EDS) and dynamic light scattering (DLS) [21]. Specifically, the XRD is used for the phase identification and determination of the crystalline structure of the prepared nanoparticles [46]. The morphological features such as shape and size are easily studied using TEM and SEM at the nanometre to micrometre scale [91], whilst the surface charge and the size distribution of the nanoparticles suspended in a liquid are studied using DLS. The optical properties of these materials could be studied using some spectrophotometric techniques, such as UV–visible and fluorescence spectrophotometers [92]. The EDS measures the elemental composition of the material, while FTIR is useful in the characterization of the surface chemistry by identifying the functional groups attached to the surface of the nanoparticles [21,46,93]. Thus, upon establishing the possible properties of the nanoparticles using these characterization techniques, the desired application could then be explored.

To this end, a modified summary adapted from the review report of Lin et al. [87], which shows a concise collection of various physiochemical characterizations for bionanomaterials with their respective advantages and disadvantages, has been presented in Table 2.

## 3. Biological Importance of Biogenic Metal-Based Nanoparticles

About 60% of commercially available drugs are either directly or indirectly derived from natural sources, such as plants, animals, and minerals [94]. This thus creates a platform in which these medicinal plants can be carefully selected in such a way that a synergistic biological activity can be imposed on the desired biological system, thereby offering an extra advantage in their usage as mediating agents for the synthesis of biocompatible nanomaterials. The used metallic component of biogenic metal-based nanoparticles (MNPs) also provides for a large surface area and multiple oxidation states, which in turn allows for high reactivity [95]. This green synthetic route involving the use of natural products, therefore, offers a tremendous comparative advantage over other regular therapeutic agents, such as slow drug release in the biological system, increasing half-life, and improving efficacy as is the case with those used for chemotherapeutic purposes [96]. Hence, there are now new nanomedicines that have been optimized for improved drug absorption, distribution, metabolism, excretion, and less toxicity (ADMET) [97]. For instance, silver nanoparticles have been reported to show significant levels of toxicity when administered orally [98], but their synthesis, using the active ingredients from natural sources, has been reported to participate in the particulate formation of MNPs and the adsorption of unique chemical entities onto the particle surface, which in turn enhances biocompatibility, stability, biological activities, and reduced toxicity [99]. Moreso, using medicinal plant resources for nanoparticle synthesis offers the advantages of availability of raw materials, cost-effectiveness, and ease of mass production [100]. These MNPs have thus been reported to show several biological activities, such as antioxidant, anti-inflammatory, antimicrobial, antiviral, and anticancer activities.

### 3.1. Plant-Mediated Metal-Based Nanoparticles as Antimicrobial Agents

Multi-drug resistant (MDR) pathogenic microorganisms have become a serious issue and increasingly a public health problem. This is because of the rising cases of microbial infections and infectious diseases worldwide, as well as the difficulties in achieving and sustaining adequate concentrations of tissue antibiotics while limiting systemic drug exposure to tolerable levels [101,102]. Methicillin-resistant *staphylococcus aureus* (MRSA) is an example of a prevalent MDR bacterium that has successfully transitioned from an almost exclusively nosocomial setting to being capable of causing a disease epidemic [103]. Therefore, new strategies that are more effective, less toxic, and affordable antimicrobial drugs (antibiotics) are desired. “Nanobiotics” (NBs), which entail the application of nanotechnology for the development of antibiotics, are gradually becoming a major driving force behind recent changes in antimicrobial drug discovery [102]. This class of antibiotics ensures the sustained release of active drugs by a novel mechanism of synchronous drug delivery; thus, making them more effective and a better choice than the traditional antibiotics in recent times [102]. With the introduction of NBs in the last two decades, they offer the advantages of drug solubilization, reduced toxicity, sustained release, increased efficacy, and improved pharmacokinetics and biocompatibility [104].

**Figure 7:**
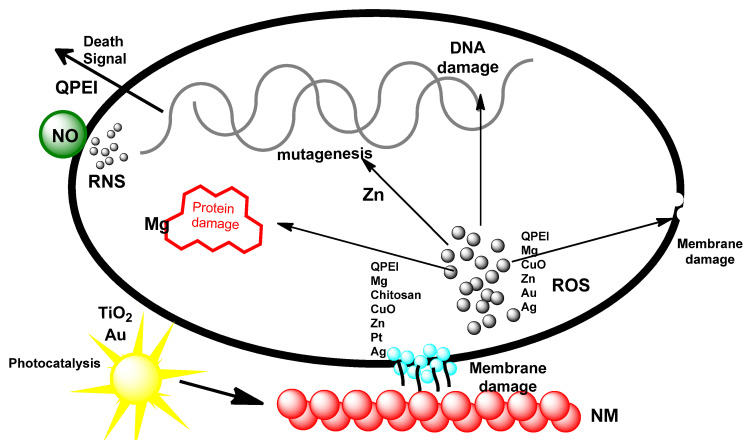
General schematic representation of the antibacterial mode of action for metal-based nanoparticles. The MNPs interact with the membrane of the bacteria, causing membrane disruption. This interaction also brings about the frequent generation of free radicals (ROS yellow spots) which may generate another secondary damage, hinder protein function, cause DNA destruction, and result in excess radical production. These nanomaterials can also proceed via photoactivation (photocatalyst); nitric oxide (NO) NM is involved with RNS (green spots). Redrawn from [107], with permission from Hindawi Publishing Corporation (copyright 2022).

In recent years, concerns have been raised over the effect of nanoparticle-based antibiotics which are of synthetic origin on human health and the environment [105] Consequently, these have resulted in the alternative use of natural products because they provide antimicrobial surfaces that tend to be non-toxic and eco-friendly. For instance, catechin, a natural antibacterial flavonoid, is a popular biogenic component involved in plant-mediated metal-based nanoparticles [105]. Ordinarily, MNPs are unstable, and easy oxidation has limited their use as an antibacterial agent. However, in recent times and utilizing biogenic routes, metal nanoparticles such as the novel “Catechin-Cu-Nanoparticles” have been found to be stable and capable of targeting the pathogenic bacteria, *Escherichia coli*, *Staphylococcus aureus* and their multi-drug resistant (MDR) strain effectively [105]. Furthermore, the MDR bacteria have been reported to exhibit resistance to many biogenic MNPs more slowly than to commercial small-molecule drugs [106], and this makes them very useful in the fight against antimicrobial resistance. The MNPs with antimicrobial properties generally thus act simultaneously along two major lethal pathways, which are the disruption of membrane potential and integrity, and the production of reactive oxygen species (oxygenated free radicals), in which these materials act as nanocatalysts [107] (see Figure 7 for the mode of action). Other reports on the antimicrobial properties of many metals, metal oxide, and bimetallic nanoparticles have been summarized in Table 3.

### 3.2. Plant Mediated Metal-Based Nanoparticles as Anticancer Agents

Cancer otherwise known as neoplasia or malignant tumor is currently among the leading causes of death worldwide, accounting for about 10 million mortalities in the year 2020 alone [157]. Cancer has been projected to reach about 26 million morbidities and could claim up to about 17 million lives by the year 2030 if not well managed [158]. The advent of genomics, proteomics, and bioinformatics has revealed the complexities of cancer [159]. Despite efforts to reduce the burden of cancer through radiotherapy, immunotherapy, surgery, hormone therapy, targeted therapy, hyperthermia, photodynamic therapy, stem cell transplant, and chemotherapy, cancer remains an incurable and one of the deadliest diseases [160]. The challenges to the effective treatment of cancer have been thought to include the metastatic nature of cancer, its stem cell viability, numerous onco-types, and drug specificity to the different cancer types and/or cancer cell lines [161]. Conventional cancer chemotherapy is plagued with the inability to penetrate and reach the core of solid tumors, failing to kill the cancerous cells, and non-selective action to the cancerous cells only; thereby, resulting in side effects such as myelosuppression, mucositis, thrombocytopenia, alopecia (hair loss), and organ dysfunction [162]. These side effects could lead to delay in treatment, dose reduction, or discontinuance of the given drugs [162]. The discovery of some biogenic anticancer agents, such as vincristine and vinblastine from the Madagascar Periwinkle (*Catharanthus roseus*), and paclitaxel commercially known as taxol from *Taxus brevifolia*, have provided more insight and instigated more interest in the role of nature, especially medicinal plants in cancer chemotherapy [163]. Many plant extracts are known to be cytotoxic to specific cancer types and non-toxic to normal human cell with little or no side effect unlike their synthetic counterparts [163,164]. They have been thought to become new platform for the continuous discovery of useful anticancer drugs. Nevertheless, this effort is believed to be plagued by the complexities associated with the treatment of different cancer type, drug specificity to cancer types, and the difficulty to achieve optimal therapeutic value [163,164]. Recent technological advances such as the application of nanotechnology in medicine (nanomedicine) are having a profound impact on cancer diagnosis, treatment, and monitoring [165]. It is a technique that ensures direct access of nanoparticles to cancerous cells selectively, with increased drug localization, cellular uptake, accurate drug delivery, and non-interference with the healthy cells [162]. This platform offers improved cancer drug delivery through increased solubility and sustained retention time. This technique also allows for conjugation of nanoparticles with tumor-specific ligands; thus, improving drug delivery and efficacy with a significant reduction in toxicity [166]. Biogenic metal-based nanoparticles (MNPs) are among the group of nanoparticles (NPs) that are currently being explored in cancer drug delivery due to their well-known advantages such as ease of photosynthesis, eco-friendliness, biocompatibility, reliability, and cost [167]. The uniqueness of the physicochemical properties of metal-based nanomaterials makes them an interesting research tool in cancer, such as radiotherapy, gene therapy, cellular bioimaging, tumor detection, and targeted drug therapy [112]. The metallic component of nanoparticles can be functionalized with various molecules such as monoclonal antibodies, transferrin, and anticancer drug, to produce nanocarriers. These nanocarriers, such as those of Au-NPs, have shown potential for drug delivery to targeted sites by crossing the blood-brain barrier in the treatment of brain tumors [150]. 

Furthermore, literature studies have shown that the size of a nanoparticle plays a role in its level of cytotoxicity amongst other biological properties [168]. For instance, the biogenic gold nanoparticles (AuNPs), with a particle size of about 2.0 nm showed significant cytotoxicity due to their ability to enter the cell nucleus. However, upon the increase in size to about 10 nm, the cytotoxicity against the same cancer cells was reported to be significantly reduced [168]. Biogenic metal-based nanoparticles thus have excellent immunogenicity and modifiability which in turn helps in the transportation of tumor therapeutic drugs to achieve combined therapy, with improved effectiveness and durability of antitumor immunity while still reducing adverse side effects [169]. Other reports on plant-mediated metal NPs with some cytotoxicity/anticancer activities have been summarized in Table 3.

### 3.3. Plant Mediated Metal-Based Nanoparticles as Antioxidant Agents 

Antioxidants are basically chemical substances that delay or prevent the release of free radicals or counter the free radical released into the biological system. These free radicals are generated by reactive species, capable of causing oxidative or nitrosative stress, which result to different disease conditions [170]. Under normal conditions, the human body produces antioxidants to counteract the ill effects of free radicals. However, during the shortage of these natural antioxidants in the body, a need is created which is usually supported by external sources [171]. Example of notable substance that has been used includes butylated hydroxyanisole (BHA) and butylated hydroxytoluene (BHT). Despite their extensive usage, these substances have exhibited adverse effects, which include hepatotoxicity and carcinogenicity [171]. Conversely, natural antioxidants such as *L-*ascorbic acid, gallic acid, β-carotene, α-tocopherol, lycopene, resveratrol, and quercetin are non-toxic; hence, their preferential biomedical use [172]. 

The application of MNPs as antioxidant agents of choice is fast emerging as a novel contender in biomedicine due to the implicated synergistic interaction between natural antioxidants and MNPs in the entire nano-synthesized entities [173]. So far, biogenic nanoparticle-based antioxidants are proving to be the new biomedical tool for the effective management of disease morbidity and mortality. Documentary evidence has shown the prospect of MNPs as useful antioxidant agents [17,76,174,175,176]. They have been reported to offer better activities than those of plant extracts alone, as well as when compared to their MNP counterparts, which is partly due to the reducing and stabilizing actions of the natural product contents, as well as their role as capping agents in the nanostructure [115]. Singh et al. [177] showed the uniqueness in the antioxidant activity of MNP in the green synthesis of silver-based nanoparticles, mediated using the whole flower bud extract of *Couroupita guianensis*. The reducing potentials of the natural products (plant extracts, fractions, constituents) component of MNPs has been reported to be responsible for the observed antioxidant potentials of many MNPs [116]. The antioxidant potentials of MNPs by implication connote varying degrees of biological activities, including anti-inflammatory, antibacterial, antifungal, antiviral, and anticancer activities, amongst others. Other reports of their antioxidant activities have been reported in Table 3.

### 3.4. Plant Mediated Metal-Based Nanoparticles as Anti-Inflammatory Agents 

There has been an increasing prevalence of chronic inflammation in the last few decades, due to factors that include unhealthy lifestyle (poor diet, alcoholism, and tobacco smoking), exposure to occupational hazards (poisonous chemicals), and the emergence of multi-drug resistant pathogens [178]. Inflammation is a biological response of the immune system to toxic or harmful triggers, such as irradiation, toxic compounds, damaged cells, or pathogens [179]. it acts by removing the injurious triggers and initiating the healing process; inflammation is, therefore, a biological defense mechanism [179]. These inflammations can either be acute or chronic, which may, in turn, induce mild or severe inflammatory responses in the lung, liver, kidney, pancreas, intestinal tract, reproductive system, heart, and brain [180]. The clinical consequences of chronic inflammation could be severe and include pathogenic infections, liver cirrhosis, chronic kidney diseases, hypertension, cardiovascular diseases, hyperglycemia, increased risk of metabolic syndrome, various types of cancer, depression, neurodegenerative and autoimmune diseases [181]. 

**Figure 8:**
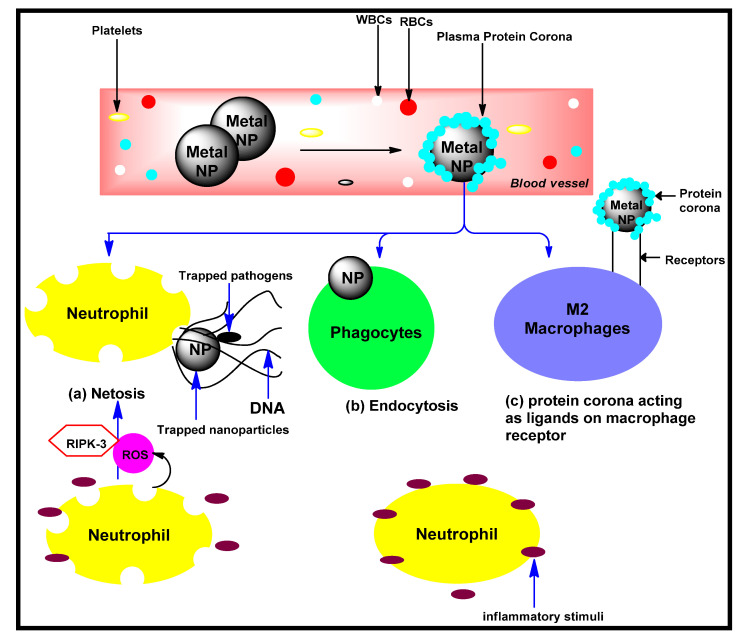
The anti-inflammatory mechanism adopted by metal-based nanoparticles. Redrawn from [187], with permission from Elsevier (copyright 2022).

Anti-inflammatory drugs are essential in the treatment regimens of many diseases and are among the most consumed types of drugs in the world [182]. Several conventional drugs classified as steroidal anti-inflammatory drugs (SAIDs) and non-steroidal anti-inflammatory drugs (NSAIDs) have been developed. The NSAIDs are more common and are grouped according to their chemical structures and selectivity. They include acetylated salicylates (aspirin), non-acetylated salicylates, propionic acids (acetic acids, ibuprofen, diclofenac, indomethacin), enolic acids (meloxicam, piroxicam), anthranilic acids (meclofenamate, mefenamic acid), and selective cyclo-oxygenase 2 inhibitors (celecoxib and etoricoxib) [183,184]. Unfortunately, these drugs have not been able to achieve the best condition of efficacy and safety, due to their serious side effects and compounded health problems [183,184]. Nanomedicine has thus emerged as a novel strategy and as a powerful therapeutic alternative, for the effective and safe treatment of inflammations. Nanoparticles allow for increased delivery, active accumulation, and controlled drug release into the diseased cells and tissues, thereby reducing toxicity and side effects [185]. It is on this backdrop that many nanomedicines were developed to treat inflammatory-related diseases, such as chronic wounds, microbial infections, cancer, cardiovascular and neurodegenerative diseases, as well as metabolic syndrome [185]. Thus, the biological potentials of MNPs derived from medicinal plants can be attributed to the effect of functional groups attached to them, and the nano range of their sizes. For instance, the anti-rheumatic (anti-inflammatory) effect of Selenium nanoparticles (SeNPs) has been reported, and it was considerable due to its potential in ameliorating oxidative stress-mediated inflammation via downregulation of radical and nonradical species, markers of inflammation, and the upregulation of inherent antioxidant defenses [186]. Details of the mechanism involved in the anti-inflammatory properties of metal-based nanomaterial is summarized in Figure 8. Other reports of their anti-inflammatory activities have been reported in Table 3.

### 3.5. Plant Mediated Metal-Based Nanoparticles as Antiviral Agents

Viruses are microorganisms of either DNA or RNA genetic codes, covered by protein coats. They multiply by first infecting other biological cells of humans, animals, or other lower organisms, and then use components of the host cell to replicate [103]. They have become a serious public health problem and are responsible for many diseases such as HIV/AIDS, Measles, Ebola, Influenza, Polio, Smallpox, and more recently and the novel severe acute respiratory syndrome coronavirus 2 (SARS-CoV-2) infection [188]. To date, vaccination remains the best way to prevent viral infections. Unfortunately, viruses mutate at a very alarming rate, rendering many of the available vaccines ineffective [188], which has thus emphasized the need to develop more effective and safe antiviral drugs. The development of an antiviral drug that will not adversely affect the host is challenging, because of dependence of the host machinery for viral replication, limitations of in vitro assays, and low availability of in vivo assay methods capable of simulating human viral infections [189]. Natural products, especially medicinal plants offer an inexhaustible armoury of bioactive ingredients that could be developed as new antiviral agents. Recently, indirubin and indigo in the leaves and fruits of *Couroupita guianensis* were reported to inhibit the major protease (Mpro) of COVID-19 and other coronaviruses [190]. More recently is the application of nature-inspired nanoparticles (NPs), for efficient antiviral drug delivery [191]. Nanotechnology has changed the face of viral research. It has been used in the design of biosensors and bioelectronics for virus detection [111]. Moreover, the versatility of NPs makes them considered a powerful tool for the prevention and treatment of viral infections, because of their unique physical and chemical properties that can be exploited and controlled in the process of synthesis [192]. So far, nanotechnology has yielded remarkable results such as the quasi-spherical silver nanoparticles with an aqueous extract of *Panax ginseng* roots, which was found to be significantly virucidal against influenza A virus [113]. Likewise, silver-nanoparticles synthesized using plant extract of *Lampranthus coccineus* and *Malephora lutea* have demonstrated considerable antiviral activities (HAV-10, HSV-1, CoxB4) [130]. 

A table summarizing different types of metal-based nanoparticles synthesized using constituents of plant materials alongside some plant-like microorganisms and the probable active ingredient responsible for the reduction and stabilization of these nanoparticles as well as their respective biological potentials have been presented in Table 3

## 4. Challenges and Future Prospects

There is an increasing interest to improve drug delivery for the resolution of diseases. This interest thus represents a unique opportunity for candidates like biogenic metal-based nanoparticles with improved biodistribution and pharmacokinetics, [185]. Nature-inspired metallic nanoparticles represent a new generation of innovative nanomedicines designed to mimic natural circulatory cells [162]. These materials have been found to have the capacity to increase blood circulation time and improve the distribution of the loaded drug towards cells and tissues [162]. The contribution of nanotechnology in the precise treatment of diseases, which are often with lesser life-threatening side effects, can potentially contribute to the positive movement in clinical practice for life-saving approaches [162]. However, their immunogenicity, scale-up, and characterization remain important hurdles during clinical trials [193]. Besides the problems related to the scaling-up, government regulations and the overall cost-effectiveness in comparison to the currently available chemotherapies are other important limitations in the success of nanomedicines. The often-complex architectural design of many BMNPs may also likely result in difficulties for performing reproducible sample preparations, safe and in good quantities. Their reproducibility has been identified as one of the greatest challenges as a slight modification of the size, the shape, and/or the nanoparticle surface chemistry may dramatically influence the stability, the interaction with biological media, as well as their biodistribution. Thus, reliable, and standardized methodologies to obtain reproducible nanoparticles are required. Furthermore, the gap between the laboratory, where innovative materials are designed, and the industrial replication of the process, where reproducible preparation and manufacturing processes are carried out, has to be narrowed for these materials to be excellent biological tools [193].

Nevertheless, despite the highlighted drawbacks, it is exciting that many discoveries are fast emerging in nanotechnology. Amongst such is the design of some unique nanoparticles that communicate with cancer cells [194]. This discovery could lead to novel, drug-free therapeutics that can slow and/or stop cancerous growth. Perhaps, the much-awaited future of safe and effective drug delivery is here.

## 5. Conclusions

The use of eco-friendly methods, such as those involving plant extracts, in the preparation of metal-based nanoparticles, has proven to be a useful route over the years, due to ease of preparation, eco-friendliness, and the biocompatible nature of the prepared material with most biological systems. These advantages have been attributed to the presence of some useful phytochemicals (with medicinal benefits) and other materials within the plants. Many of these refined benefits have been thought to emerge due to the changes in some notable properties of the nanomaterials such as size, shape, and optical properties. As highlighted in this review, many active ingredients of some plants have thus been effectively used to reduce different metal ions to their respective metal-nanoparticles, whilst also stabilizing them and conferring useful properties, including biological activities, from the plants on such synthesized nanoparticles. This, in turn, has created an alternative pathway for synthesizing useful therapeutic agents in the fight against microorganisms and their resistance, cancer, and excessive production of free radicals in the body. Their importance in medicine is therefore very significant as already indicated, regardless of some unwanted drawbacks. These nanosized therapeutic agents have already shown extreme effectiveness at the cellular level for all kinds of diseases. Consequently, further research into how they can be safely used could lead to novel therapeutic pathways.

## Figures and Tables

**Table 1:** Examples of metal-based nanoparticles synthesized using plant extracts.

Type of MNPs	Conditions	Properties	Plants	Refs.
Ag Au	25 to 95 °C	15 to 50 nm, cubic 5 to 40 nm, spherical	*Ginkgo biloba* leaves,	[54] [55]
Au	25 °C	~100 nm spherical	*Mirabilis jalapa* flowers	[56]
Au	25 °C	5–85 irregular, rod shape	*Avena sativa* stem	[57]
Ag	25 °C	35 nm, triangular	*Pinus thunbergii*	[58]
Au	25 to 95 °C	15 to 25 nm, spherical	*Trigonella-foenum graecum* seeds	[59]
Ag	25 to 95 °C	40 nm spherical	*Alternanthera sessilis* whole plant	[60]
Ag	30 to 95 °C	13 to 27 nm spherical	*Andrographis paniculata* leaves	[61]
Ag Ag, Au	37 °C	20 to 30 nm, spherical 20 to 30 nm, spherical	*Acalypha indica* leaves	[62] [63]
Ag Au/Ag	95 ^o^C	15 to 90 nm spherical 50 to 500 nm cubic	*Diospyros kaki*	[64]
Ag/Au	25 °C	50 nm	*Swietenia mahogani* leaves	[65]
Cu	25	20 to 110 spherical	*Euphorbia esula* leaves	[66]
Pb Pt	80 °C	16 to 20 nm, spherical 6 to 8 nm, irregular	*Pinus resinosa* bark	[67]
Fe^2+^ Ag	25 to 95 °C	50 nm spherical	*Sorghum* bran	[68]
In_2_O_3_	60°C	5 to 50 nm, spherical	*Aloe vera* leaves	[69]
TiO_2_	60 °C	100 to 150 nm spherical	*Annona squamosa* peel	[70]
ZnO	60 °C	5 to 40 nm Spherical	*Calotropis procera*	[71]
NiO	60 °C	16 to 52 nm spherical 11 to 59 nm	*Zingiber officinale* (ginger) *Allium sativum* (garlic)	[72]
CuO	75 °C	4.8 nm spherical	*Sterculia urens*	[2]

**Table 2:** Some analytical techniques for the physiochemical characterizations for nanomaterials with their respective advantages and disadvantages. Redrawn from [87], with permission from Elsevier (Copyright 2022).

Physiochemical Properties	Analytical Technique	Advantages	Disadvantages
Phase, size, shape, and structure of crystalline materials	XRD	Widely recognized technique. Resolutions at the atomic scale are very spatial.	Limited to only crystalline materials. In comparison to electron diffraction, possess low intensity. Only a single conformation/binding state of the sample is accessible.
Structure and conformation of bioconjugate surface properties	Infrared spectroscopy (IR). Attenuated total reflection Fourier transform infrared (ATR–FTIR)	Cheap and Fast measurement. Modern ones (ATR-FTIR) require no sample preparation, which makes them easily reproducible. Regardless of sample thickness, measurement can be acquired.	The older version may possess complicated sample preparation (IR) procedure. Interference and strong absorbance of H_2_O (IR). Sensitivity may be low at the nanoscale.
Hydrodynamic size distribution.	Dynamic light scattering (DLS)	Measurement can be achieved in any solvent of interest. Results are easily reproducible. Materials can be easily collected after analysis (non-destructive and invasive). For monodisperse samples, their hydrodynamic sizes are accurately measured. Equipment is not too expensive.	Measurement is influenced by small numbers of large particles. For polydisperse samples, measurements are limited. Size resolutions are limited. Techniques assume that all samples are spherical. Insensitive size correlations.
Stability referring to surface charge	Zeta potential	Many samples can be measured simultaneously.	Measurements are not easily reproducible. Electro-osmotic effect.
Aggregation/agglomeration Dispersion Shape Size and distribution	Scanning electron microscopy (SEM) Environmental SEM (ESEM)	Images of material are obtained in high resolutions. Possibilities for direct size measurement and their distribution. The size of the material can be easily observed. Biomolecules in their natural state can be easily captured using ESEM.	Only dry samples are required. Sample analysis is in non-physiological conditions (except ESEM). In a heterogeneous sample, there is potential for a biased statistic in the allocation of size distribution. The instrument is very expensive.
Aggregation/agglomeration Dispersion Shape Size and distribution	Transmission electron microscopy (TEM)	The shape of material with higher spatial resolution than SEM can be easily observed and measured. Size and size distribution can be directly measured.	Very thin sample is required in non-physiological conditions. Possibility for poor sampling, and damage. Equipment is expensive.
Chemical and electronic properties. Hydrodynamic size and size distribution (indirect analysis). Conformation changes of protein–metallic NP conjugate structural.	Raman scattering (RS) Surface-enhanced Raman (SERS) Tip-enhanced Raman spectroscopy (TERS)	Does not require sample preparation. Increases spatial resolution (SERS). Gives topological information (SERS, TERS). Potential for detecting tissue abnormality. Enhanced RS signal (SERS).	Measurements are not reproducible. Fluorescence interferences. Cross-sections are extremely small. Resolutions are limited. Signals are weak compared to Rayleigh scattering.
Hydrodynamic dimension Binding kinetics.	Fluorescence correlation spectroscopy (FCS)	Many particles can be measured simultaneously (using ELS). Can study chemical kinetics, molecular diffusion, the effect of concentration, and conformation dynamics. Possess high spatial and temporal resolution. Uses up small samples for fluorescent probes.	Limit in fluorophore species. Limited applications and inaccuracy due to a lack of appropriate models.
Size/size distribution Shape Structure	Small-angle X-ray scattering (SAXS)	Simple sample preparation is required. Non-destructive method. Amorphous samples can be easily measured.	-
Aggregation/agglomeration Dispersion Shape Size and distribution Surface properties (Modified AFM)	Atomic force microscopy (AFM)	Mapping of the sample surface in 3D. Direct measurement of samples in aqueous, ambient, and dry environments. Sub-nanoscale topographic resolution.	Lateral dimensions are usually overestimated. Time-consuming. Poor sampling. Only exterior properties are measured.

**Table 3:** Some Plant mediated Metal-based Nanoparticles with their Biological Potentials.

Biological Source	Natural Extract/Compound	Type of MNPs	Biological Activity	Reference
Plant	Catechin	CuO-NPs	Antibacterial	[105]
	Almond seed extract	AuNPs-Quercetin AuNPs-Camptothecin	Anti-inflammatory, anticancer, anti-angiogenic	[108]
	Fruit extract of *Couroupita guianensis* Aubl.	AuNPs	Antioxidant	[109]
	Extract of *Taraxacum officinale* leaf	AgNPs	Antioxidant, anticancer, antimicrobial	[110]
	Extract of *Ocimum sanctum* leaf	AuNPs	Antioxidant, reducing ability	[111]
	Dragon fruit from the genus *Hylocereus*	AuNPs	Anticancer (Breast cancer)	[112]
	Extract of *Panax ginseng* root	AgNPs	Anticancer, antiviral	[113]
	Extract of *Lantana camara* leaf	AgNPs	Antibacterial, catalytic	[114]
	*Cissus quadrangularis*	AgNPs	Antimicrobial, Larvicidal, Cytotoxicity	[115]
	Extracts of *Rosmarinus* sp. and *Zataria multiflora* aerial parts	AgNPs	Antioxidant and reducing capacities	[116]
	Extract of *Cyclopia intermedia*	AuNP	Anticancer	[117]
	Extract of *Curcuma longa* rhizomes	AuNP-conjugated graphene oxide	Antioxidant and anticancer	[118]
	Aqueous extract of *Melia azedarach* leaf	AgNPs	Antioxidant, antibacterial, wound healing effect, antidiabetic	[119]
	*Punica granatum* leaf extract	AgNPs	Antidiabetic and anticancer	[120]
	*Perilla frutescens* leaf extract	AgNPs	Antioxidant, antibacterial and anticancer	[121]
	*Arisaema flavum* tuber extract	AgNPs	Antibacterial	[122]
	*Citrus clementina* peel extract	AgNPs	Antimicrobial, anticancer	[123]
	*Pisum sativum* outer peel aqueous extract	AgNPs	Antidiabetic, anticancer, antioxidant, antibacterial	[124]
	*Aesculus hippocastanum*	AgNPs	Antibacterial, antioxidant, drug release system	[125]
	Fruit extract of *Limonia acidissima* and conjugated epirubicin	AuNPs	Targeted drug delivery against breast cancer	[126]
	*Sargassum incisifolium* Aqueous Extracts	AgNPs AuNPs	Antimicrobial, anticancer	[127]
	*Mukia maderaspatna* fresh leaf extract	AuNPs AgNPs	Anticancer	[128]
	*Cinnamomum cassia*	AgNPs	Antiviral	[129]
	*Lampranthus coccineus* and *Malephora lutea*	AgNPs	Antiviral	[130]
	Seed extract of *Embelia ribes*	AuNPs AgNPs	Antioxidant, antimicrobial, anticancer	[131]
	Extract of *Anacardium occidentale*	AuNPs	Cytotoxic (breast cancer)	[112]
	Extract of *Lycium chinensis*	AuNPs	Anticancer	[132]
	Dried fruit extract of *Amomum villosum*	AuNPs	Antioxidant, antimicrobial, anticancer	[133]
	Fruit extracts of *Aegle marmelos, Eugenia jambolana,* and *Soursop*	AuNPs	Anticancer	[134]
	Podophyllotoxin extract from *Linum usitassimum*	AuNPs	Anticancer	[135]
	Xanthone derivative (mangiferin) from *Mangifera indica* leaves	AuNPs	Non-toxic to normal human breast cell line	[136]
	*Citrus macroptera*	AuNPs	Anticancer	[112]
	Kaempferol glucoside from *Lotus leguminosae*	AuNPs	Antioxidant, anticancer	[137]
	Aqueous fruit extract of *Chaenomeles sinensis*	AuNPs, AgNPs	Antioxidant, antimicrobial, anticancer	[138]
	*Syzygium aromaticum*	AgNps	Antiviral	[139]
	*Backhousia citriodora* leaf extract	AuNPs	Antioxidant, anticancer	[140]
	*Corchorus olitorius* extract	AuNPs	Anticancer	[141]
	Aqueous root extract of *Glycyrrhiza uralensis*	AuNPs AgCl-NPs	Antimicrobial, antioxidant, anticancer	[142]
	*Olea europaea* leaf extract	CuO-NPs	Anticancer, non-toxicity to normal cells	[143]
	*Ficus religiosa* leaf extract	CuO-NPs	Anticancer	[144]
	Leaf extracts of *Cissus quadrangularis* and *Piper betle*	CuO-NPs	Antibacterial	[145]
	leaf extracts of olive (*Olea europaea*)	ZnO-NPs	Antioxidant	[146]
	*Falcaria vulgaris* leaf extract	CuO-NPs	Anticancer, antioxidant, antifungal, antibacterial, cutaneous wound healing	[147]
	Aqueous extracts of aerial roots of *Rhaphidophora aurea* intertwined over *Lawsonia inermis* and *Areca catechu*	AuNPs	Anticancer	[148]
	*Cotyledon orbiculata* fresh leaf extract	AgNPs	Anti-inflammatory	[149]
	*Ganoderma lucidum* -oriental Mushroom extract	AuNP-Doxorubicin conjugate	Anticancer	[150]
	*Nigella arvensis* leaf extract	AuNPs	Antibacterial, antioxidant, anticancer, catalytic	[151]
Marine plants	Seaweed (*Sargassum wightii*) extract	AgNPs	Antibacterial	[152]
	Seaweed (*Gelidiella acerosa*) extract	AgNPs	Antifungal	[153]
	Carrageenan oligosaccharide derived from marine red alga	AuNPs	Antitumour	[154]
	*Penicillium fellutanum*	AgNPs	Antimicrobial	[155]
Algae	n-hexane and ethyl acetate fractions of *Nannochloropsis* sp.	AgNPs	Antioxidant, antimicrobial, anticancer	[156]
	*Dunaliella salina*	AuNPs	Anticancer (Breast cancer)	[112]

key: AuNPs—Gold nanoparticles; AgNPs—Silver nanoparticles; AgCl-NPs—silver chloride nanoparticles; CuO-NPs—copper oxide nanoparticles; ZnO-NPs—zinc oxide nanoparticles; SeNPs—selenium nanoparticles.

## Data Availability

Not applicable.
